# Very-early-onset autoimmune hypothyroidism: a report of two cases with STAT3 gain-of-function variant

**DOI:** 10.1530/ETJ-25-0260

**Published:** 2025-12-08

**Authors:** Rowmika Ravi, Anita Niskanen, Mary Pat Reeve, Kristiina Makkonen, Harri Niinikoski, Jorma Toppari, Jukka Kero

**Affiliations:** ^1^ Department of Clinical Sciences, Faculty of Medicine, University of Turku, Turku, Finland; ^2^ Institute for Molecular Medicine Finland, HiLIFE, University of Helsinki, Helsinki, Finland; ^3^ Department of Paediatrics and Adolescent Medicine, Turku University Hospital, Turku, Finland; ^4^ Institute of Biomedicine, Research Centre for Integrative Physiology and Pharmacology, and Centre for Population Health Research, and InFLAMES Research Flagship Centre, University of Turku, Turku, Finland

**Keywords:** early-onset autoimmune hypothyroidism, *STAT3*, genetic testing, thyroid disorders

## Abstract

Autoimmune hypothyroidism occurs rarely before 3 years of age. Two siblings were diagnosed with autoimmune hypothyroidism at age 5 and 16 months, presenting with classic symptoms of hypothyroidism, abnormal thyroid function tests (TSH: 200 and 660 mU/L; reference range (RR): 0.73–8.4 mU/L; Free T4: 5.9 and <1.3 pmol/L; RR: 11.9–25.6 pmol/L), and high thyroid peroxidase antibody levels. Thyroxine medication alleviated their symptoms. Apart from mild infections, the siblings exhibited no other major disorders. Whole exome sequencing identified a pathogenic *STAT3* gain-of-function variant, most commonly associated with infantile-onset multi-organ autoimmune disorder. Genetic testing for early-onset hypothyroidism may reveal specific etiologies, impacting follow-up and treatment.

## Established facts

- Autoimmune hypothyroidism rarely occurs under 3 years of age and is commonly accompanied by other autoimmune manifestations.
- *STAT3* gain-of-function variant is associated with early-onset multisystem autoimmune disease.

## Novel insight

- *STAT3* gain-of-function patients could atypically manifest very-early-onset autoimmune hypothyroidism as the first and only major autoimmune disorder.

## Introduction

Autoimmune hypothyroidism (AIH) is a common cause of thyroid dysfunction, most frequently diagnosed in adulthood. AIH occurring under 3 years of age is very rare (1, 2) and may be associated with monogenic poly-autoimmune disorders, typically featuring severe multi-organ manifestations such as lymphoproliferation, enteropathy, or type 1 diabetes, involving variants in genes such as *FOXP3* or *STAT3* (3). Here, we report a case of very-early-onset AIH in two otherwise healthy siblings, which prompted genetic analysis and led to the identification of a known *STAT3* pathogenic variant.

## Case presentation

### Study subjects

Participants were recruited by a pediatric endocrinologist at Turku University Hospital. Written informed consent was obtained from all participants or their legal guardians. Detailed methods are mentioned in the Supplementary data (see section on Supplementary materials given at the end of the article).

### Patient 1a

**Figure 1:**
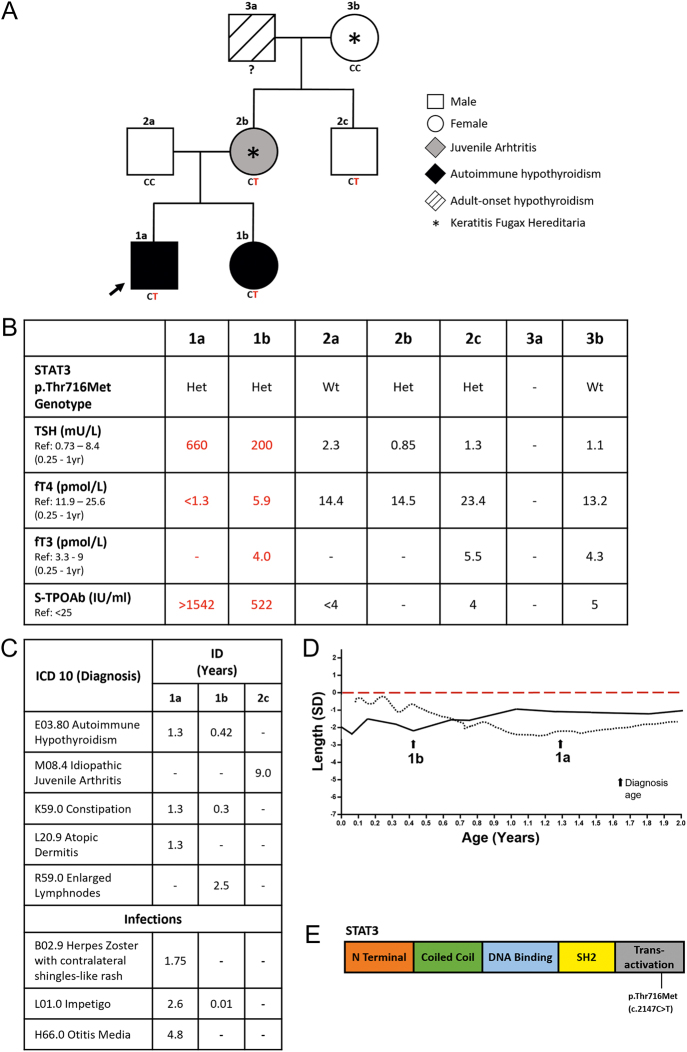
(A) Pedigree of the two siblings with very-early-onset autoimmune hypothyroidism (AIH) and *STAT3* gain-of-function (GOF) variant. *STAT3* p.Thr716Met genotypes are indicated as CT (heterozygous) and CC (wild type). Abnormal TFT values in the affected siblings (1a and 1b) are highlighted in red. (B) Table presenting serum TSH, fT4, fT3, and TPO antibody (TPOAb) test results. Values for patients 1a and 1b were measured before initiation of thyroxine replacement therapy. (C) Table showing the diagnoses (ICD-10 codes) and ages at diagnosis (in years) for the affected individuals. (D) Growth curves of the affected patients (1a and 1b). Arrowheads indicate ages at diagnosis. Growth normalized in both siblings following standard thyroxine treatment. (E) Schematic representation of the STAT3 protein, highlighting the location of the pathogenic variant p.Thr716Met in the trans-activation domain.

A Finnish boy was born prematurely at 30 + 6 weeks of gestation (birth weight 1.625 g (+0.2 SD), length 40.2 cm (−0.5 SD), head circumference 30.8 cm (+2.1 SD), Apgar 9/9/10) by C-section due to abnormal heart sounds (shown in Fig. 1A). Newborn screening for congenital hypothyroidism (CH) was normal (umbilical TSH: 6.2 mU/L, reference range (RR): <40). During infancy, he exhibited accelerated linear growth up to approximately 12 months of age, after which growth deceleration became evident. Around this time, symptoms such as constipation, poor appetite, and delayed motor development emerged. At 16 months, thyroid function tests (TFTs) revealed elevated thyrotropin (TSH), low free thyroxine (fT4), and high thyroid peroxidase antibody (TPOAb) titers (shown in Fig. 1B), leading to a diagnosis of primary autoimmune hypothyroidism (AIH) and thyroxine replacement therapy. Thyroid ultrasound showed a slightly thickened isthmus and a more heterogeneous echotexture than normal, suggestive of early thyroid inflammation, but without clear evidence of goiter (shown in Supplementary Fig. S1). Treatment resulted in marked improvement in symptoms and growth velocity, as illustrated in the growth chart (shown in Fig. 1D). He lacked the HLA-DQB1*02 and DQB1*0302 alleles associated with increased autoimmune risk. Other investigations were unremarkable (shown in Supplementary Table S1). At 8 years old, apart from a history of varicella-zoster infection and mild atopic eczema, he had no other major clinical manifestations (shown in Fig. 1C).

### Patient 1b

Patient 1a’s sister was born at 37 + 1 weeks (2.926 g (−0.6 SD), 47 cm (−0.8 SD), head circumference 32.5 cm (−1.1 SD), Apgar 9/10/10). Newborn screening for CH was normal (TSH 5.8 mU/L, RR: <40). At 5 months, TFTs were performed due to constipation, poor appetite, sleepiness, and slight growth delay. AIH was diagnosed, and thyroxine was started (shown in Fig. 1B). Thyroid ultrasound at 6 months showed normal echotexture and lobe sizes (right lobe AP: 10 mm, left lobe AP: 7 mm) without focal lesions. A follow-up ultrasound at the age of 3 years revealed a small and heterogeneous right lobe, with more heterogeneous tissue in the left lobe and isthmus, consistent with inflammatory changes typical of autoimmune thyroiditis, but without discrete nodules. Treatment rapidly improved her symptoms and growth (shown in Fig. 1D and Supplementary Fig. S1). Other test results were normal (shown in Supplementary Table S1). At age 4, she had no signs of other autoimmune diseases.

### Family members

The mother (2b) had HLA-B27–negative juvenile idiopathic arthritis (JIA) with minimal symptoms and has had no medication in adulthood (shown in Fig. 1A). She also had prolonged leukocytosis and multiple dermatofibromas. Both she and her mother (3b) had iritis (shown in Fig. 1C). The maternal grandfather (3a) had adult-onset hypothyroidism. The father (2a) and maternal uncle (2c) are healthy (shown in Fig. 1A and B).

## Genetic analysis

Whole-exome sequencing identified a known monoallelic *STAT3* gain-of-function (GOF) variant c.2147C>T, p.Thr716Met (3) in the affected siblings (1a, 1b), their mother (2b), and her brother (2c) (shown in Fig. 1A). The p.Thr716Met pathogenic variant is very rare and is neither present in gnomAD (https://gnomad.broadinstitute.org/) nor the FinnGen database (https://www.finngen.fi/en). The ‘iritis’ observed in 2b and 3b was attributed to a pathogenic *NLRP3* variant (c.61G>C, p.Asp21His), associated with Finnish keratitis fugax hereditaria (KFH) (4). As KFH shares overlapping features with iritis, genetic testing was warranted. No other pathogenic variants in thyroid-specific or autoimmune-related genes were identified. Genetic results were validated using Sanger sequencing (shown in Supplementary Fig. S2 and Supplementary Table S2). Variants of uncertain significance are shown in Supplementary Table S3.

## Discussion

Very-early-onset AIH before 3 years of age is rare (1, 2) and may suggest a monogenic etiology. We describe a family with severe AIH in two siblings, diagnosed at 5 and 16 months of age, carrying a known rare *STAT3* GOF variant, but without other apparent phenotypes during the follow-up.

STAT3 encodes a transcription factor involved in signaling pathways of several ligands, including interferons, interleukin (IL)-5, and IL-6, regulating proliferation, differentiation, and immunosuppression (3). The most common manifestations of STAT3 GOF syndrome include lymphoproliferation, autoimmune cytopenia, enteropathy, and other autoimmune disorders (5). Given that *STAT3* GOF variants usually cause multiple severe features, isolated AIH is atypical (shown in Supplementary Fig. S3) (5).

The presence of the p.Thr716Met pathogenic variant in the mother, who has JIA, and in the asymptomatic uncle (2c), suggests incomplete penetrance and variable expressivity. Although no other manifestations have appeared, future disease risk warrants continued monitoring. Screening for blood count abnormalities, diabetes- and celiac-related autoantibodies may aid in early detection of type 1 diabetes, lymphopenia, or enteropathy. Patients with *STAT3* GOF variants have been treated with IL-6 or JAK inhibitors (5), which could be considered if new severe autoimmune manifestations emerge.

In conclusion, we report very-early-onset AIH as the first and only major autoimmune manifestation in two *STAT3* GOF patients. Genetic testing for early-onset hypothyroidism may uncover specific etiologies, with implications for follow-up and treatment.

## Supplementary materials



## Declaration of interest

The authors declare that there is no conflict of interest that could be perceived as prejudicing the impartiality of the work reported.

## Funding

This study was supported by the Finnish Paediatric Society (Grant number 190001), the Sigrid Jusélius Foundation (Grant number 115-1956-26), and the Novo Nordisk Foundation (Grant number 0078329).

## Patient consent

Written informed consent was obtained from the patients' legal guardians for publication of the submitted article and accompanying images.

## Author contribution statement

RR and JK conceptualized the study. RR performed the sequencing and interpretation of the data. MPR evaluated the FinnGen data. JK, JT, HN, and KM recruited and characterized the clinical data. RR, JK, and AN wrote the draft of the manuscript, and all authors contributed to the writing and editing of the final version.

## Statement of ethics

The study was approved by the Ethics Committee of the Hospital District of Southwest Finland (108/180/2010) and conducted complying with the Declaration of Helsinki (2024).

## References

[1] Foley TP, Abbassi V, Copelnad KC, et al. Brief report: hypothyroidism caused by chronic autoimmune thyroiditis in very young infants. N Engl J Med 1994 330 466–468. (10.1056/NEJM199402173300704) 8289852

[2] Marzuillo P, Grandone A, Perrotta S, et al. Very early onset of autoimmune thyroiditis in a toddler with severe hypothyroidism presentation: a case report. Ital J Pediatr 2016 18 42–61. (10.1186/s13052-016-0270-7) PMC4912771 27316517

[3] Fabre A, Marchal S, Barlogis V, et al. Clinical aspects of STAT3 gain-of-function germline mutations: a systematic review. J Allergy Clin Immunol Pract 2019 7 1958–1969. (10.1016/j.jaip.2019.02.018) 30825606

[4] Immonen AT, Kawan S, Vesaluoma M, et al. Clinical spectrum and geographic distribution of keratitis fugax hereditaria caused by the pathogenic variant c.61G>C in NLRP3. Am J Ophthalmol 2022 236 309–318. (10.1016/j.ajo.2021.10.025) 34740632

[5] Leiding JW, Vogel TP, Santarlas VGJ, et al. Monogenic early-onset lymphoproliferation and autoimmunity: natural history of STAT3 gain-of-function syndrome. J Allergy Clin Immunol 2023 151 1081–1095. (10.1016/j.jaci.2022.09.002) 36228738 PMC10081938

