# Supplementary material for: Very-early-onset autoimmune hypothyroidism: a report of two cases with STAT3 gain-of-function variant
Source: Eur Thyroid J. 2025 Dec 8;14(6):e250260. doi: 10.1530/ETJ-25-0260 (PMC12687928; doi:10.1530/ETJ-25-0260)
Supplement: Supplementary file 1 [file supplementary_materials.pdf]

Supplementary Appendix

Supplementary Materials and Methods

Laboratory measurements

The clinical examinations were performed by a pediatrician and thyroid ultrasound by the pediatric radiologists. Laboratory tests were done at the Turku University Hospital Laboratory. Umbilical serum TSH, serum TSH, fT4 concentrations were determined with the Cobas e801 immunoassay analyzer (Roche Diagnostics, Germany). Growth data were collected from hospital records.

Genetic analysis

DNA was isolated from 5ml of peripheral blood using kit and protocol provided by the manufacturer (Qiagen, Valencia, CA). Whole exome sequencing was performed for the siblings and the parents using a commercial service (Novogene Co., Ltd). The variant was validated and tested in other family members by Sanger sequencing. PCR was performed by standard protocol (Thermo Fisher Scientific, Waltham, MA USA) Primer sequences are listed in Supplementary Table S2. Commercially available genetic variant analysis platform (AION v3.12.0.1, Nostos Genomics GmbH) was used to identify pathogenic/likely-pathogenic variants according to ACMG/AMP 2015 guidelines. Alamut Visual Plus v1.9 (© 2023 SOPHiA GENETICS) was used to visualize the Sanger sequences.

FinnGen

The FinnGen data release 12 was used for evaluation. In the FinnGen database, there were no individuals born after 2019 that were tested for anti-TPO antibodies at < 1.5 years of age. The FinnGen project has been approved by the Ethical Review Board of the Hospital District of Helsinki and Uusimaa with the protocol Nr. HUS/990/2017. Detail information of the different releases is described in the FinnGen's website.

Supplementary Figures

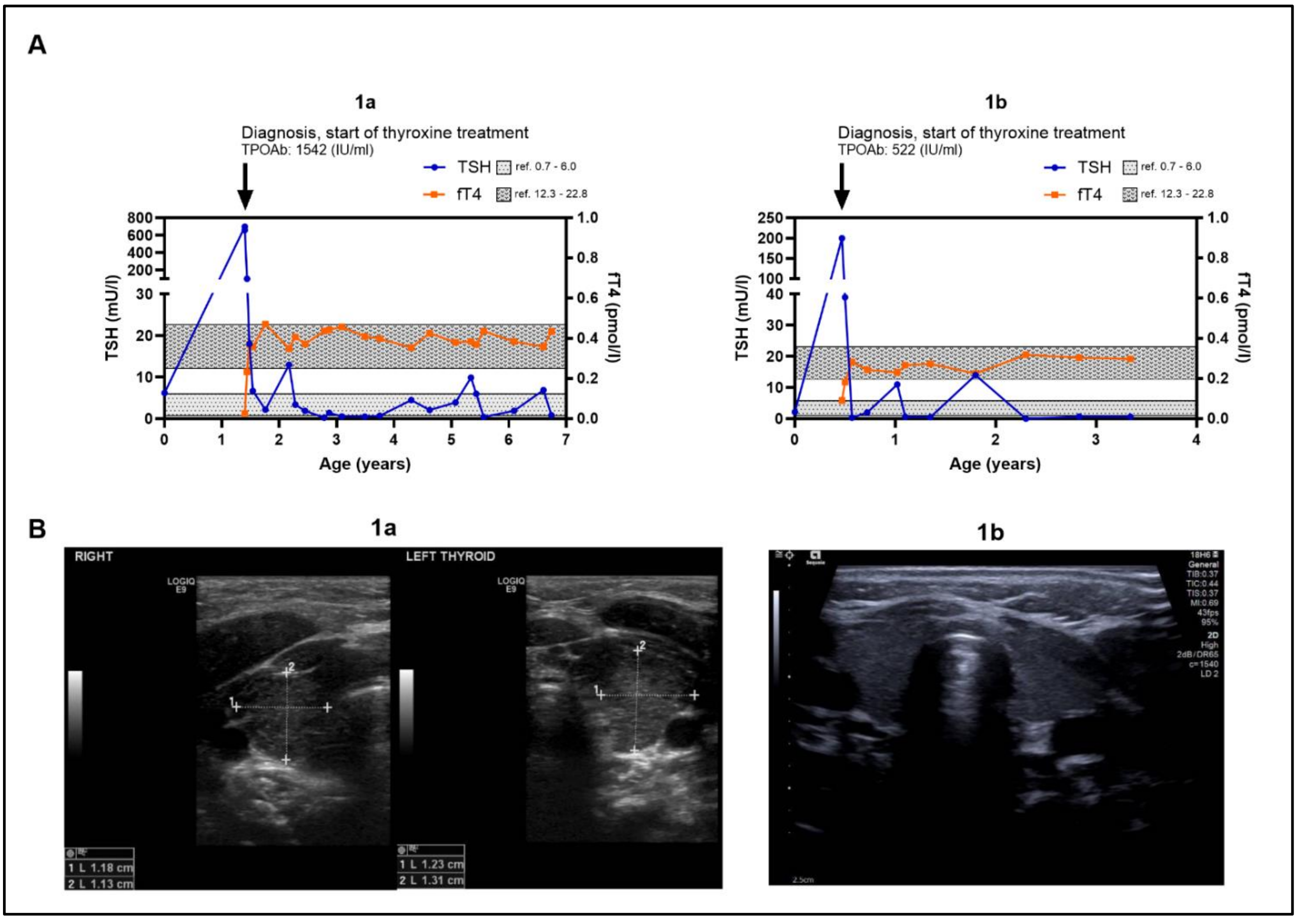

Figure S1. Panel A: Serum thyrotropin (TSH), free thyroxin (fT4,) and thyroid peroxidase antibody (TPOAb) concentrations of patients 1a and 1b with reference range. **Panel B**: Thyroid ultrasound images of patients 1a and 1b. Patient 1a shows slight inflammation with an otherwise normal thyroid gland at 16 months of age. Patient 1b shows a smaller left thyroid lobe due to autoimmune destruction at five months of age.

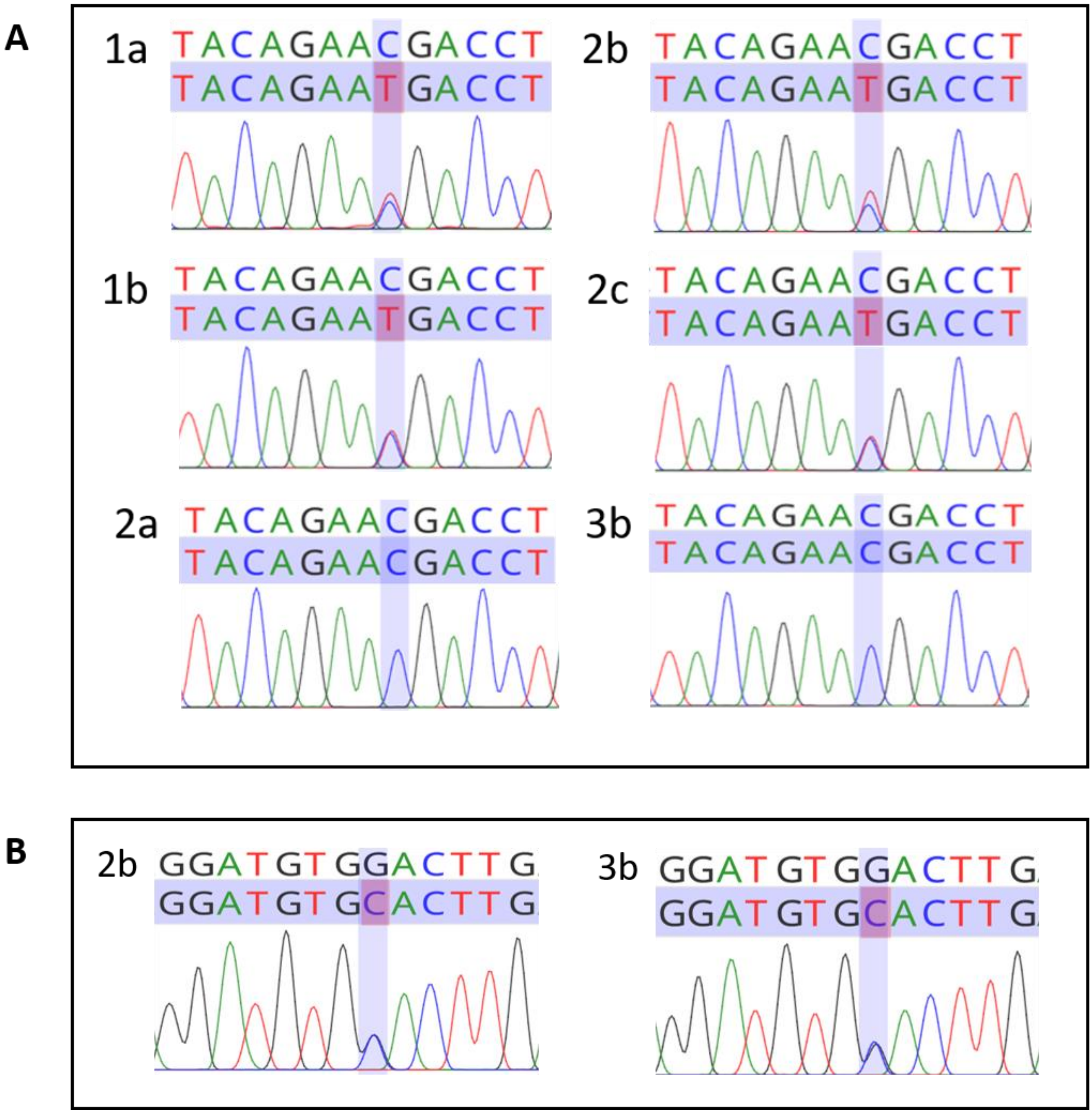

Figure S2. Panel A: Sanger sequencing chromatograms of the *STAT3* locus in the index and the family members. Heterozygous variation (CT) and wildtype (CC) position is highlighted. **Panel B**: Sanger sequencing chromatograms of the *NLRP3* locus in the family members affected with Keratitis Fugax Hereditaria. The position of the heterozygous variation (GC) is highlighted.

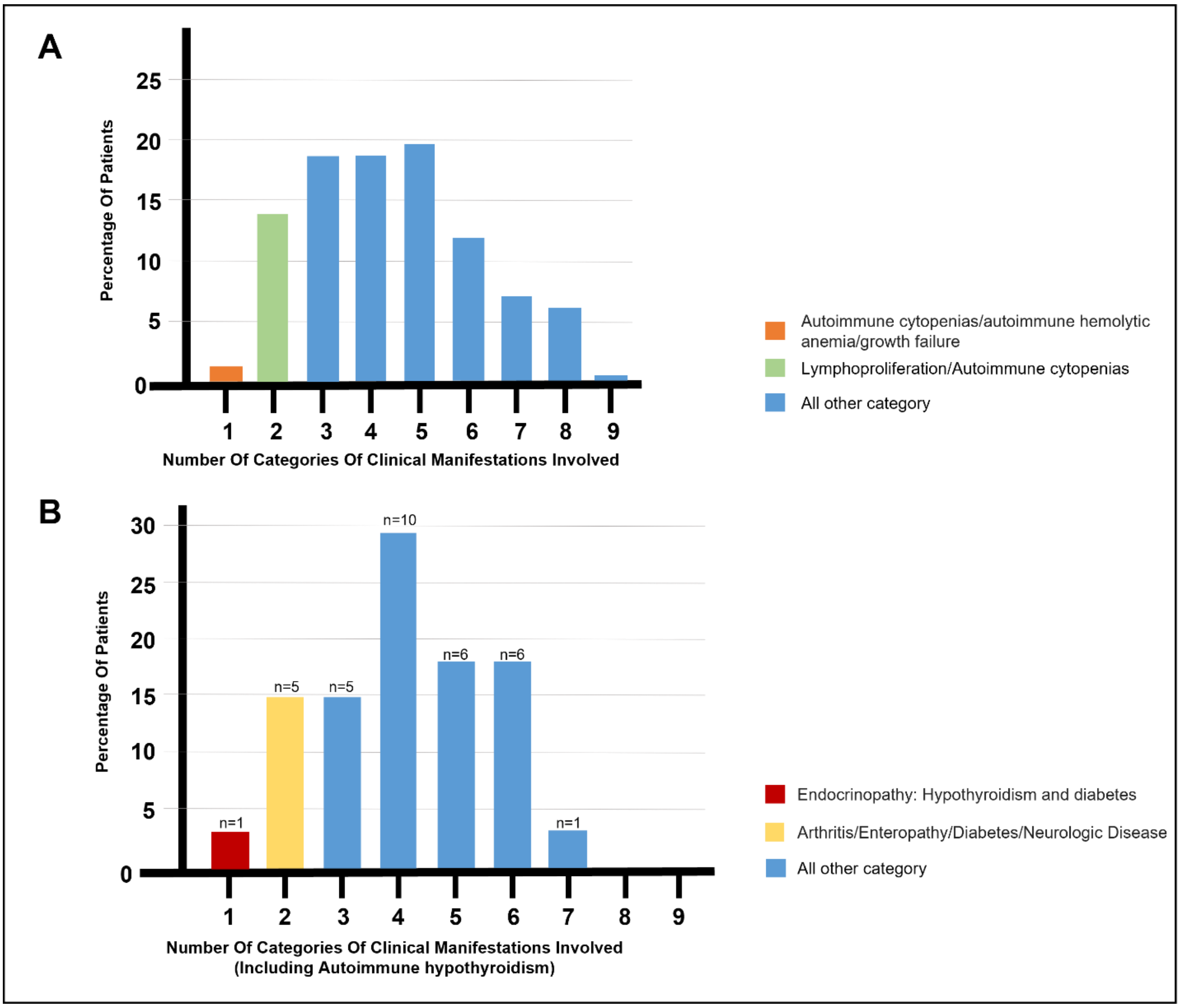

Figure S3. Clinical manifestations of STAT3 GOF patients. **Panel A**: Percentage of each clinical manifestation within the 191 patients (Leiding *et al*). **Panel B**: Percentage of each clinical manifestation within the 34 patients who had autoimmune hypothyroidism. Graph generated with data obtained from Leiding *et al.* Figure depicts the rare event of organ-specific clinical manifestation. Only one patient out of 34 had one category of clinical manifestation involving hypothyroidism and diabetes. None of the patients were affected with autoimmune hypothyroidism alone.

Supplementary Tables

Table S1: Tests/screenings with normal results in patients 1a and 1b during follow-up

S.No	Test/Screening	Patient ID
1	Basic Blood count	1a, 1b
2	Complement activity	1a, 1b
3	B and T- cell amount and distribution	1a, 1b
4	Toll-like receptors	1a
5	Kidney function	1a
6	Liver function	1a
7	Vitamin D	1a
8	Calcium	1a
9	Celiac disease	1a, 1b
10	Type 1 Diabetes	1a, 1b
11	Adrenal Autoantibody	1a, 1b
12	Bayley's testing	1a
13	Head MRI	1a
14	Vision Testing	1a
15	Glutamate decarboxylase	1b

Table S2: List of primers used for Sanger sequencing

Target site	Forward primer	Reverse primer
*STAT3*_p.Thr716Met	CGAGTGACCAGCTCTCGGTGT	GTTGGAAGCCCCTGGGCTATGT
*NLRP3*_p.Asp21His	CCGTGTTCACTGCCTGGTAT	TCTCTCCTGTTGATCGCAGC

Annealing temperature of the primer pairs was 60°C.

Table S3: Additional disease relevant variants identified in exome sequencing data, classified as benign or variant of uncertain significance.

Gene	ID	Chr location (GRCh38.p13)	Protein change	rsID	Genotype	gnomAD MAF	Clinvar	ACMG
TG	1a, 1b, 2a	8:132869751	p.Gly67Ser	rs116340633	Het	0.008791	B	LB
THRB	1a, 1b, 2a	3:24190202	p.Glu52Gly	rs775495128	Het	0.00001301	.	VUS
NFKB1	1a, 1b, 2b	4:102579000	p.Arg231Cys	rs558348827	Het	0.00001921	VUS	VUS
DUOX2	1b, 2a	15:45101227	p.Phe966SerfsTer29	rs530719719	Het	0.002839	P/LP	LP
